# A Rare Case of Multi-Organ Complications Due to Disseminated Citrobacter koseri Infection Originating From a Renal Abscess

**DOI:** 10.7759/cureus.110813

**Published:** 2026-06-14

**Authors:** Sara Pinto-Magalhães, Raquel Pinto, Sara Lino, João Gonçalves, Maria José Manata

**Affiliations:** 1 Infectious Diseases, Unidade Local de Saúde de São José, Lisboa, PRT; 2 General Surgery, Unidade Local de Saúde de São José, Lisboa, PRT

**Keywords:** antibiotherapy, citrobacter koseri, diabetes mellitus, perirenal abscess, septic embolism, source control

## Abstract

*Citrobacter koseri* is a gram-negative bacillus that can cause severe infections in immunocompromised individuals. While it is a rare urinary tract pathogen, its potential for dissemination and severe complications demands attention. We report the case of a 44-year-old male with poorly controlled type 2 diabetes mellitus who developed a disseminated *C. koseri* infection originating from a perirenal abscess. The infection extended locally, resulting in septic thrombosis of the left renal vein, and spread distantly, causing septic pulmonary embolism. The patient underwent multiple surgical interventions, including left nephrectomy and Hartmann’s procedure, in addition to prolonged antimicrobial therapy. Despite persistent complications, including an intra-abdominal abscess with cutaneous fistulization and iatrogenic hydropneumothorax, a conservative management approach with antibiotic therapy ultimately led to clinical resolution. This case underscores the importance of early recognition, appropriate source control, and individualized antimicrobial strategies, particularly in patients with complex infections and multiple comorbidities.

## Introduction

*Citrobacter koseri* is a gram-negative, facultatively anaerobic bacillus that belongs to the *Enterobacteriaceae* family. It is an opportunistic pathogen capable of causing various infections, particularly in infants, older adults, and immunocompromised individuals [1]. In neonates, *C. koseri *is classically associated with meningitis and brain abscesses, which may result in significant morbidity and mortality [2]. In adults, it is associated with infections affecting the respiratory system, urinary tract, intra-abdominal organs, skin, and soft tissues [3,4].

Several risk factors predispose individuals to *C. koseri* infections, including structural abnormalities of the urinary tract, diabetes, malignancies, and other conditions that compromise the immune system [3-5].

Although renal abscesses caused by *C. koseri* are uncommon, they may lead to severe clinical consequences if not promptly treated. Untreated infections may spread through the bloodstream to other organs, potentially leading to complications such as pneumonia, endocarditis, and endogenous endophthalmitis, among others. Locally, the infection can cause renal abscess and fibrosis, which may eventually progress to chronic kidney disease [3,5].

## Case presentation

The patient was a 44-year-old man, originally from India, who had been living in Portugal for approximately one year. He had a medical history of type 2 diabetes mellitus, treated with metformin 1000 mg daily. In 2024, he presented to the emergency department with a two-week history of general malaise, fatigue, dry cough, nausea, vomiting, and left flank abdominal pain. Upon physical examination, the patient had a fever, decreased breath sounds at the left lung base, and tenderness on palpation of the left abdominal quadrants, without signs of peritoneal irritation. The laboratory findings revealed anemia, leukocytosis with neutrophilia and lymphopenia, elevated C-reactive protein, renal function impairment, hyponatremia, hyperkalemia, and marked hyperglycemia with ketosis and elevated serum osmolality, in the absence of metabolic acidosis, consistent with hyperglycemic hyperosmolar syndrome (Table 1). Screening for human immunodeficiency virus and hepatitis B and C was negative.

**Table 1:** Laboratory results at the time of hospital admission.

Variable	Reference Range	At admission
pH	7.35 - 7.45	7.42
HCO_3_ (mmol/L)	22.0 - 26.0	23.9
Lactate (mmol/L)	0.5 - 1.6	1.6
Glucose (mg/dL)	60 - 100	635
Ketonemia (mmol/L)	< 0.6	3.5
Hemoglobin (g/dL)	13.0 - 17.0	7.1
White-cell count (cells/μL)	4500 - 11000	15420
Neutrophil count (cells/μL)	2000 - 8500	13990
Lymphocyte count (cells/μL)	900 - 3500	640
C-reactive protein (mg/L)	< 5.0	221.8
Creatinine (mg/dL)	0.67 - 1.17	1.64
Urea (mg/dL)	16.6 - 48.5	78.0
Sodium (mEq/L)	136 - 145	123
Potassium (mEq/L)	3.5 - 5.1	5.8
Serum osmolality (mOsm/Kg)	275 - 295	321
Glycated hemoglobin (%)	< 5.7	14.9
Albumin (g/L)	35.0 - 52.0	11.8

A computed tomography (CT) scan of the chest, abdomen, and pelvis revealed multiple peripheral pulmonary nodules, some cavitary, suggestive of septic embolization (Figure 1); bilateral pleural effusion, more pronounced on the left; a left renal abscess (41 × 29 × 122 mm) extending into the perirenal space, psoas muscle, and anterior pararenal space (Figure 2), with absent contrast uptake by the left kidney, suggesting loss of renal function; complete, non-acute thrombosis of the left renal vein, considered septic in the setting of the adjacent renal abscess; and thickening of the descending colon wall, possibly secondary to local extension of the infection.

**Figure 1:**
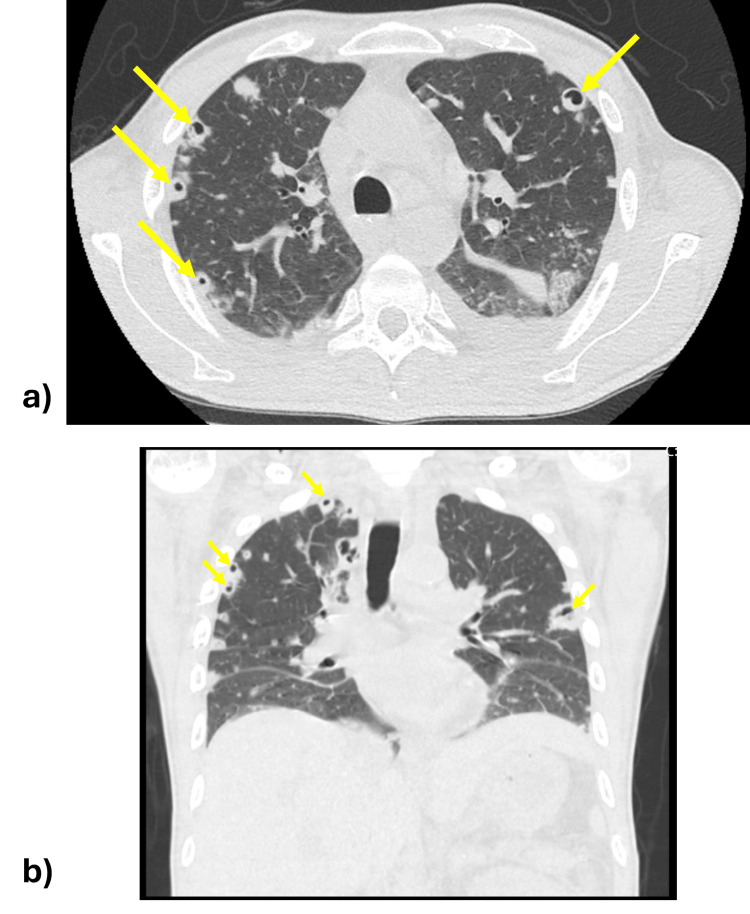
Thoracic CT scan: axial (a) and coronal (b) planes. Yellow arrows indicate cavitated lesions consistent with septic pulmonary embolism. CT: computed tomography

**Figure 2:**
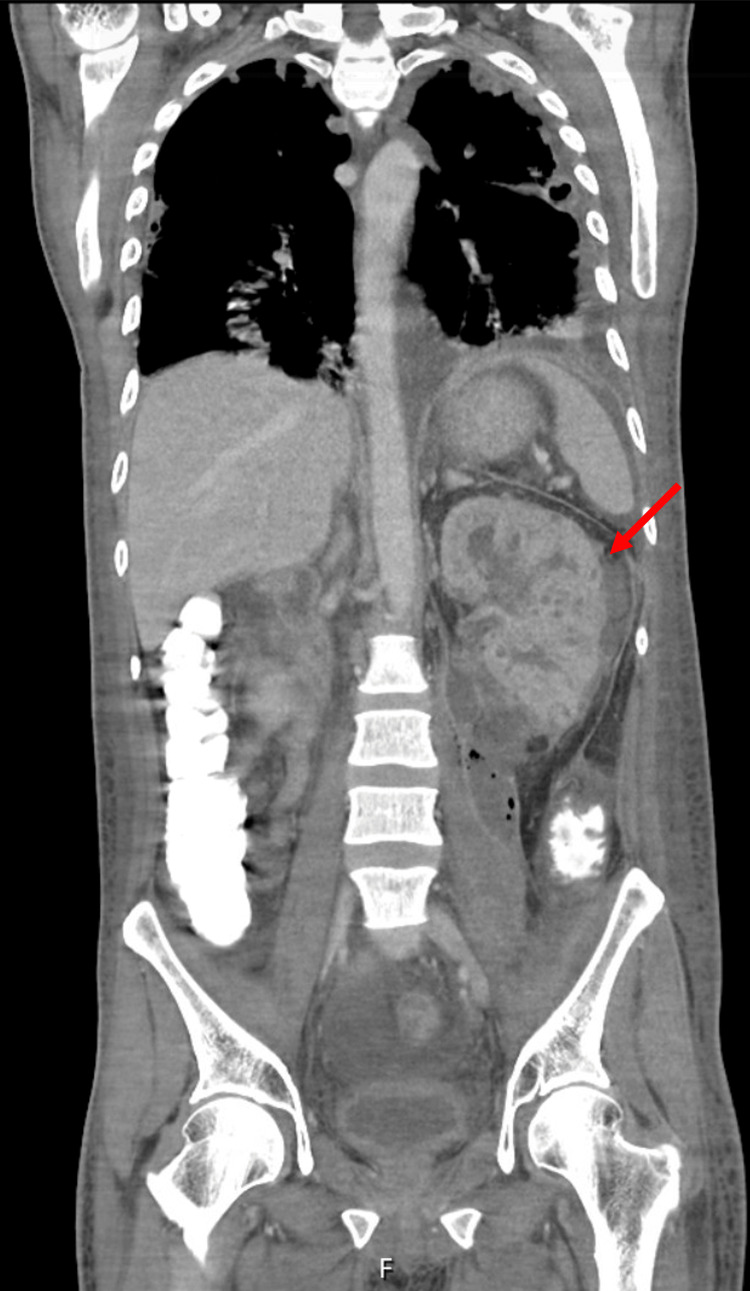
Abdominopelvic CT scan: coronal plane. The red arrow indicates a left renal abscess extending into the perirenal space, psoas muscle, and anterior pararenal space.

The patient was started on empirical piperacillin/tazobactam therapy and subsequently admitted to the Infectious Diseases Department for further management. A timeline summarizing the patient's clinical course during hospitalization is presented in Figure 3.

**Figure 3:**
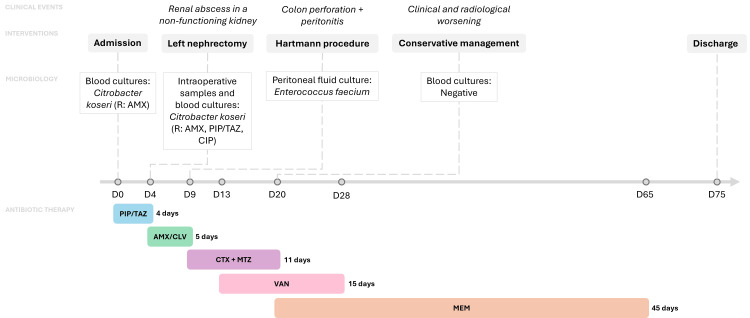
Timeline of clinical events, microbiological findings, and antibiotic therapy during hospitalization. AMX: Amoxicillin; AMX/CLV: Amoxicillin/clavulanic acid; CIP: Ciprofloxacin; CTX + MTZ: Ceftriaxone + Metronidazole; MEM: Meropenem; PIP/TAZ: Piperacillin/tazobactam; VAN: Vancomycin

On the fourth day of hospitalization, a left nephrectomy was performed for source control of infection in a non-functioning kidney. During the procedure, the pleura was inadvertently breached, resulting in drainage of a purulent pleural effusion and subsequent iatrogenic left hydropneumothorax. Based on the antimicrobial susceptibility profile of *C. koseri* isolated from blood cultures collected at admission (susceptible to amoxicillin/clavulanic acid, cefotaxime, and gentamicin, and resistant to ampicillin/amoxicillin), antibiotic therapy was switched to amoxicillin/clavulanic acid. Pleural fluid and renal abscess cavity fluid were collected intraoperatively, and a new set of blood cultures was obtained on the same day. *C. koseri* was subsequently isolated from all samples, exhibiting a different antimicrobial susceptibility profile, including resistance to piperacillin/tazobactam and ciprofloxacin.

An abdominal and pelvic CT scan was repeated a few days after surgery, revealing a perforation of the descending colon adjacent to the previous renal abscess site, along with secondary peritonitis. The patient underwent Hartmann's procedure, including left colectomy and terminal colostomy. Following the colonic perforation, antibiotic therapy was changed to ceftriaxone and metronidazole. Vancomycin was subsequently added after isolation of *Enterococcus faecium* (susceptible to tigecycline and vancomycin) from peritoneal fluid collected during surgery.

Despite repeated attempts at source control, including two major surgical interventions and drainage of both surgical sites, the patient developed an intra-abdominal abscess with secondary cutaneous fistulization. Given his poor clinical condition due to the severity of the infection and multiple iatrogenic complications, a conservative approach was adopted, consisting of spontaneous drainage of the intra-abdominal abscess through the cutaneous fistula and systemic antibiotic therapy. The patient completed 11 days of ceftriaxone and metronidazole and 15 days of vancomycin. However, due to clinical worsening, supported by increasing inflammatory markers and radiological progression of the pulmonary involvement on computed tomography, the antibiotic regimen was escalated to meropenem, and a chest drain was placed to manage the left hydropneumothorax. Repeat blood cultures were obtained and remained negative. Given the pulmonary lesions consistent with septic embolization, transthoracic and transesophageal echocardiography were performed to evaluate for endocarditis, which was ultimately excluded.

The patient completed 45 days of meropenem, corresponding to a total of 56 days of antibiotic therapy following the last source control intervention (Hartmann's surgery), with good clinical and imaging responses in both the pulmonary and intra-abdominal involvements and no adverse events related to meropenem therapy. At discharge, laboratory evaluation demonstrated improvement of the patient's nutritional status, with serum albumin increasing to 25.2 g/L. Renal function had also normalized despite the previous nephrectomy, with a serum creatinine of 0.92 mg/dL and an estimated glomerular filtration rate of 100 mL/min/1.73 m². He was discharged after 75 days of hospitalization, functionally independent, and was referred to a general surgery consultation for subsequent intestinal transit reconstruction. A few weeks after discharge, the patient was lost to follow-up as he decided to return to India to recover with his family.

## Discussion

This case describes a rare presentation of disseminated *C. koseri* infection originating from a renal abscess, associated with local and distant complications.

Delayed presentation may have contributed to local progression of the infection. Furthermore, type 2 diabetes mellitus is a well-identified risk factor in similar cases reported in the literature [3-5]. The extensive renal involvement ultimately necessitated nephrectomy, underscoring the importance of early diagnosis and prompt intervention.

This case highlights that effective source control, combined with appropriate antimicrobial therapy, is fundamental in the management of severe infections and their complications [6,7]. In this case, despite multiple surgical interventions and percutaneous drainage, complete source control was not achievable. The infection progressed to an intra-abdominal abscess with cutaneous fistulization. Given the deterioration in the patient's overall clinical condition following multiple complications, a conservative approach was adopted, ultimately resulting in a favorable clinical outcome.

Defining the optimal duration of antimicrobial therapy represents an additional challenge in the absence of definitive source control. In complex intra-abdominal infections with persistent collections, treatment duration is often guided by clinical evolution and serial imaging findings rather than by robust evidence-based recommendations [8,9]. The absence of reliable biomarkers capable of determining when antimicrobial therapy no longer provides additional benefit frequently leads to prolonged treatment courses, which reflects the complexity of managing infections in the setting of incomplete source control.

An additional noteworthy finding was the change in antimicrobial susceptibility profile observed in *C. koseri* isolates recovered during hospitalization. The initial isolate, recovered from blood cultures collected at admission, differed from isolates subsequently obtained on the fourth day of hospitalization from pleural fluid, renal abscess fluid, and repeat blood cultures collected on the day of nephrectomy. Because the altered susceptibility pattern was also observed in repeat blood cultures, sampling from different anatomical compartments alone appears unlikely to explain this finding. As *C. koseri* is not typically considered an inducible AmpC-producing species [10], the underlying mechanism remains uncertain. Potential explanations include heteroresistance, selection of resistant subpopulations, or variability in antimicrobial susceptibility testing. As molecular characterization was not performed, no definitive conclusion can be drawn.

Another critical aspect of this case is the need for antibiotic selection tailored to both pathogen susceptibility and host-specific factors. Although ceftriaxone was appropriate according to susceptibility testing, severe hypoalbuminemia may alter ceftriaxone pharmacokinetics, particularly its protein binding, volume of distribution, and unbound drug exposure [11]. In the setting of high bacterial burden and incomplete source control, these factors may have contributed to a suboptimal clinical response, prompting escalation of therapy to meropenem.

## Conclusions

This case illustrates the complexities of managing disseminated *C. koseri* infection, particularly in a patient with poorly controlled diabetes mellitus. The combination of delayed medical attention, severe renal involvement, and local and distant dissemination required a multifaceted approach, including surgical intervention and prolonged antibiotic therapy. Despite aggressive management, complete source control was not achievable, requiring a conservative approach in the later stages. This case highlights the importance of early diagnosis, timely intervention, and careful antimicrobial selection. The need for individualized treatment strategies is crucial in such cases to optimize patient outcomes. Long-term outcome assessment was limited by the patient's loss to follow-up after discharge.
